# SimNet: Similarity-based network embeddings with mean commute time

**DOI:** 10.1371/journal.pone.0221172

**Published:** 2019-08-15

**Authors:** Moein Khajehnejad

**Affiliations:** Max Planck Institute for Software Systems (MPI-SWS), Saarbrücken, Germany; Instituto Nacional de Medicina Genomica, MEXICO

## Abstract

In this paper, we propose a new approach for learning node embeddings for weighted undirected networks. We perform a random walk on the network to extract the latent structural information and perform node embedding learning under a similarity-based framework. Unlike previous works, we apply a different criterion to capture the proximity information between nodes in a network, and use it for improved modeling of similarities between nodes. We show that the mean commute time (MCT) between two nodes, defined as the average time a random walker takes to reach a target node and return to the source, plays a crucial role in quantifying the actual degree of proximity between two nodes of the network. We then introduce a novel definition of a similarity matrix that is based on the pair-wise mean commute time captured, which enables us to adequately represent the connection of similar nodes. We utilize pseudoinverse of the Laplacian matrix of the graph for calculating such a proximity measure, capturing rich structural information out of the graph for learning more adequate node representations of a network. The results of different experiments on three real-world networks demonstrate that our proposed method outperforms existing related efforts in classification, clustering, visualization as well as link prediction tasks.

## 1 Introduction

Information network analysis has become ubiquitous in the past recent decades. From biology to computer sciences and from chemistry to sociology, the world is filled with networks. Building models that can effectively capture the information associated with network data has thus become increasingly important. Such models can lead to systems that are capable of performing tasks such as node ranking [1], community detection [2], classification [3] and link prediction [4].

One of the approaches towards mining graph information that recently received a significant amount of attention is the learning of graph representations, or network embeddings. The main goal of such a line of research is to learn for each node in the network a vector representation that conveys useful and meaningful information. One of the simplest approaches to learning the network embeddings can be done through the use of the adjacency matrix of the graph. For a graph of *n* nodes, the *i*-th row of the adjacency matrix corresponds to the *i*-th node, which gives us a *n*-dimensional vector representation for the node. While reasonable, one limitation with such an approach is it captures simple first-order proximity information between neighboring nodes which are directly connected to each other, ignoring higher-order proximity information amongs nodes. Furthermore, when *n* is a very large number, the dimension of the resulting embeddings become very large. Dealing with such high dimensional data becomes challenging.

Therefore it is important to develop methods that can represent the nodes in a graph with *low-dimensional* vectors, which can capture meaningful structural, semantic and relational information conveyed by the graph. Motivated by this, there has been a surge of interest in learning low-dimensional node representations for graphs in recent years. The skip-gram model [5] proposed an effective approach for learning representations for graphs with a special topology—linear chains. The model has been successfully applied to the task of learning word representations (or word embeddings) from natural language data and the model was implemented in the widely used toolkit word2vec. The model was also recently shown to have relation with an approach for learning embeddings based on factorizing a positive pairwise mutual information (PPMI) matrix [6]. DeepWalk [7], another recent work, employs *truncated random walk* to transform graph structures into linear sequences of nodes. It then makes use of the skip-gram model for learning representations for the nodes. Both of these approaches are able to capture higher *k*-th order proximity information between nodes (*i.e.*, the path between two nodes consists of *k* consecutive edges) rather than the simple first-order information as conveyed by the adjacency matrix. Such *k*-th order proximity information captures more global aspects of the network’s structure and can play a crucial role in the process of learning graph representations. In fact, in another recent study—the LINE model [8], although it largely focuses on learning local first-order and second-order proximity information, the authors also show that it is important to integrate higher-order proximity information into their model.

The importance of learning the global structural information of a graph has led to the recently proposed GraRep [9] model. In GraRep different *k*-th order proximity information between nodes is captured separately with different matrices. The model then concatenates all such information to form the low-dimensional representations for nodes. The most recent work node2vec [10] proposes to optimize a custom graph-based objective function using stochastic gradient descent motivated by [5]. The model makes use of a second-order random walk with different sampling strategies to generate neighborhood nodes for each node in the network. Next it applies the skip-gram model and optimizes the log-probability of observing a specific set of neighbors conditioned on the source node’s feature representation. The model requires the assumption that the neighboring nodes are conditionally independent of one another given the current node. We note that the above related works can all capture only the local information embedded in the network. GraRep has made an effort to gather more global information by exploring higher-order proximity information. However, the acquired global information is still bounded by the maximal length of the paths connecting two nodes.

In this paper, we propose a novel approach SimNet, which tackles the problem of learning graph representations from the perspective of measuring the global similarities between arbitrary nodes in the networks. When focusing on the network embedding approaches, one could divide them in three major groups of a. Random walk based, b. Matrix factorization based, and c. Deep learning based approaches [11]. One of the main contributions of this work is to combine the two Random walk based and Matrix factorization based approaches and use a similarity based measure to perform a highly beneficial embedding of small or large real-world networks such as DBLP network or Blocatalog datasets. Here, we first formally formulate the notion of global similarity between different two nodes based on how similar they really are. Such a similarity measure takes global structural information about the *complete network* into account, essentially involving all possible nodes rather than a local collection of neighboring nodes only. One essential component required for defining the similarities between nodes in a graph is the proximity measure between different nodes. Unfortunately, we show that existing approaches make simple assumptions when quantifying such an important measure, ignoring rich structural information conveyed by the graph. We argue that while it is true that a shorter path between two nodes indicates a higher proximity between them (and thus can lead to a higher similarity score), the length of the path connecting two nodes is not the only factor quantifying their proximity. For example, the number of possible different paths between the two nodes is also a strong factor that one needs to consider when measuring the proximity between nodes. We show that, specifically, the mean commute time (MCT) measure, among the other evaluated measures, is adequate for assessing the relative proximity between different nodes in a network when measuring their similarities. It is also demonstrated how MCT outperforms previously utilized similarity measures when exploited in the embedding process. Empirically, through extensive experiments on various datasets across different tasks, we illustrate the effectiveness of our proposed approach.

## 2 Background

In this section, we discuss background relevant to this work. We use *G* = (*V*, *E*) to denote a graph, where *V* = (*v*_1_, *v*_2_, …, *v*_*N*_) is the set of nodes and *E* = {*e*_*i*,*j*_} is the set of edges. The edge *e*_*i*,*j*_ indicates a connection between two nodes *v*_*i*_ and *v*_*j*_.

The adjacency matrix for a weighted graph is defined as a matrix *A* where [*A*]_*ij*_ = *w*_*ij*_ if and only if nodes *v*_*i*_ and *v*_*j*_ are connected by an edge with weight *w*_*ij*_ and [*A*]_*ij*_ = 0 if they are not connected by an edge. The degree of a node *v*_*i*_, denoted by *d*(*v*_*i*_), is:

$$\begin{array}{c} d\left(v_{i}\right)=\sum_{j}{\left[A\right]}_{ij} \end{array}$$ (1)

In a graph with *N* nodes the total degree of all nodes is equal to $\Sigma_{i=1}^{N}d\left(v_{i}\right)=2l$ (note that *A* is always symmetric, and *l* is the total weight of all edges in the graph) and the average degree is 2*l*/*N*. The degree matrix *D* is defined as the following diagonal matrix, where the *i*-th diagonal element is *d*(*v*_*i*_):

$$\begin{array}{c} D=diag[d\left(v_{1}\right),d\left(v_{2}\right),\ldots ,d\left(v_{N}\right)] \end{array}$$ (2)

### 2.1 Random walk

Random walk has been a subject of intensive study in the past decades. It was found useful when solving problems such as ranking [12], clustering [13, 14], synchronization [15, 16] and modeling diffusion processes [17, 18]. Today it has become an important class of probabilistic models. In this section we will briefly explain how a random walker navigates on a graph.

In a random walk, the walker currently at a node *v* can move from *v* to any of its neighbouring node with a probability proportional to the weight of the edge between them. The probability of the walker stepping into node *v*_*j*_ from *v*_*i*_ is denoted by *P*(*v*_*j*_|*v*_*i*_). Therefore the stochastic process of random walk is characterized with this transition matrix *P* which is defined as follows:

$$\begin{array}{c} P=D^{-1}A \end{array}$$ (3)

where *A* is the adjacency matrix and *D* the degree matrix defined above. Each element of *P* follows the following equation:

$$\begin{array}{c} {\left[P\right]}_{ij}=\frac{{\left[A\right]}_{ij}}{{\left[D\right]}_{ii}}=P\left(v_{j}|v_{i}\right) \end{array}$$ (4)

Let *P*^*t*^ be the *t*-th power of *P*. Then [*P*^*t*^]_*ij*_ represents the probability of the walker to arrive at node *v*_*j*_ with exactly *t* steps, starting from node *v*_*i*_.

As pointed out in [19], this kind of random walk is an example of an ergodic Markov chain [20, 21], whose stationary distribution can be characterized by a unique *N*-dimensional row vector *π* where the *i*-th element *π*_*i*_ = *d*(*v*_*i*_)/2*l*—the expected probability of reaching the *i*-th node in the random walk process.

### 2.2 Mean commute time

In this work, we would like to introduce a new method to measure the closeness or proximity between two different nodes in a network. Specifically, we introduce the mean commute time (MCT) measure to quantify this closeness. Different from many previous approaches where a simple *k*-th order proximity information is used for quantifying the closeness, we argue that MCT is a more appropriate measure whose soundness can be theoretically justified. There are two basic quantities that can be computed from the definition of the Markov chain, that is, from its transition probability matrix: the *mean first-passage time* (MFPT) [22] and then the *mean commute time* (MCT) [23, 24]. The mean first-passage time can be defined as the average number of steps (time required) that a random walker takes to reach the target node *j* for the first time starting from the source node *i*. In a close relation to MFPT, the mean commute time is defined as the average time that a random walker, starting from the source node *i*, will take to reach the target node *j* for the first time and then return to *i*. Intuitively, as it can be understood from this definition, the mean commute time between two nodes has the desirable property of decreasing when the number of paths connecting the two nodes increases and when the length of paths decreases, as opposed to the usual shortest path distance between nodes which does not capture the fact that strongly connected nodes are closer than weakly connected nodes. Therefore this quantity can give us a valuable and meaningful measure for the proximity information between nodes.

#### 2.2.1 Calculation of MCT

There are several different approaches for computing the MCT quantity based on algorithms introduced in the Markov-chain community or on iterative procedures. Here we will use the Moore-Penrose pseudoinverse of the Laplacian matrix of the graph [25] for our purpose of computing MCT.

The symmetric Laplacian matrix **L** of the graph is defined in the usual manner, **L** = *D* − *A*. Now we can introduce the Moore-Penrose pseudoinverse of **L** which will be denoted as **L**^+^ and is defined as:

$$\begin{array}{c} L^{+}={\left(L-\frac{ee^{T}}{N}\right)}^{-1}+\frac{ee^{T}}{N} \end{array}$$ (5)

where **e** is an *N*-dimensional vector consisting of all 1’s.

Pirotte *et al.* [25] showed that the MCT matrix *C* can be derived from **L**^+^ using the following procedure:

$${\left[C\right]}_{ij}\;\;=\;\;2l\left({\left[L^{+}\right]}_{ii}+{\left[L^{+}\right]}_{jj}-2{\left[L^{+}\right]}_{ij}\right)$$ (6)

$$\begin{array}{ll} & = \end{array}\;2l{\left(e_{i}-e_{j}\right)}^{T}L^{+}\left(e_{i}-e_{j}\right)$$ (7)

where **e**_*k*_ is an *N*-dimensional basis vector whose *k*-th entry is 1 and 0 elsewhere.

## 3 SimNet

### 3.1 Similarity matrix

Central to our SimNet model is the definition of a similarity matrix over the network, which captures pair-wise similarities between any two nodes in the network. The key question is how to define such a similarity matrix that is capable of capturing global similarity between two nodes. Inspired by the work of Katz *et al.* [26] which led to the definition of a type of similarity based on centrality and using the adjacency matrix [27], we use the following recursive thesis to define what is a global similarity between two nodes in a network: *Two nodes are similar to each other if one has neighbors that are similar to the other*. This statement shows that to measure the similarity between two nodes, one needs to look into the neighbors for similarity. Mathematically, we can express the above as follows:

$$\begin{array}{c} {sim}_{i,j}=\sum_{k}f\left({\left[P\right]}_{ik}{sim}_{k,j}\right)+\delta_{i,j} \end{array}$$ (8)

where *sim*_*i*,*j*_ is the similarity between nodes *i* and *j*, and *δ*_*i*,*j*_ is the indicator function which is 1 if *i* = *j* and 0 otherwise.

This expression essentially says that the similarity between two nodes *v*_*i*_ and *v*_*j*_ is defined based on a sum of functions that involve similarities defined over the neighbors of *v*_*i*_ and the node *v*_*j*_ (plus the fact whether *v*_*i*_ and *v*_*j*_ are identical). One typical choice is *f*(*x*) = *αx* where *α* < 1 (we will see why below), which leads to the following expression in matrix form:

$$\begin{array}{c} S=\alpha PS+I \end{array}$$ (9)

Repeatedly replacing the *S* in the RHS using the above recurrence relation leads to the following:

$$\begin{array}{c} S=\lim_{n\to \infty }\left(I+\alpha P+\alpha^{2}P^{2}+\cdots +\alpha^{n}P^{n}\right)=\sum_{k=0}^{\infty }g\left(k\right)P^{k} \end{array}$$ (10)

where *g*(*k*) = *α*^*k*^ is called the *proximity function*. As mentioned in [27], for the above limit to converge, *α* should have a value less than ${\left||P|\right|}_{2}^{-1}$ where ||*P*||_2_ is the spectral radius of matrix *P*. Since *P* is the normalized transition matrix, this means *α* < 1 indeed is sufficient to guarantee convergence. In fact, we have:

$$\begin{array}{c} S={\left(I-\alpha P\right)}^{-1} \end{array}$$ (11)

Now let us take a closer look at the above definition of similarity. The global similarity measure between two nodes is a combination of individual *k*-th order proximity information each augmented with a proximity function *α*^*k*^. However, what does this damping factor *α* in this function mean? Essentially it says that when calculating the current similarity matrix, how much of the similarity information captured by the neighbors we would like to rely on. A higher *α* indicates more information from the neighbors we would like to leverage on. Intuitively, it is the level of “trust” the current node would like to put to its neighbors, or how “close” a node is to its neighbors. The proximity function *g*(*k*) = *α*^*k*^ decreases exponentially as *k* increases. It was previously shown that such a function plays an important role when learning graph and word representations [5, 28–30].

Assigning a constant *α* in the above formula, we have a fixed coefficient applied to all pairs of elements of each *P*^*k*^. This means for two different pairs of nodes with the same distance, the level of closeness or proximity considered when measuring the similarities is the same regardless of their other properties or relations that can be captured by the graph. Thus using a constant *α* we have managed to see how a longer distance affects the similarity measure. Although this definition itself can allow us to capture a certain amount of global information, we believe the “closeness” between two nodes in a graph should not be defined by simply counting the number of steps required to reach one of them from the other. Instead, we believe the closeness measure should be defined in a more flexible manner, where different node pairs should be assigned a different damping factor, reflecting the true closeness between them.

This is what motivates us to use variable damping factors which are different for different node pairs, leading us to the use of MCT measure for quantifying the closeness between nodes in a graph when measuring the global similarity. The MCT between nodes contains rich structural information associated with the complete graph, which can be adequately used to measure how close two nodes are in a given graph. It is a theoretically sound measure that conveys not only the information regarding shortest path connecting two nodes, but also the number of all possible paths.

As discussed in the previous section, a lower [*C*]_*ij*_ value indicates less time required for traveling from *v*_*i*_ to *v*_*j*_, hence a higher proximity level. We thus define each entry of the *S* matrix as:

$$\begin{array}{c} {\left[S\right]}_{ij}=\sum_{k=0}^{\infty }g\left(k,i,j\right){\left[P^{k}\right]}_{ij} \end{array}$$ (12)

where *g*(*k*, *i*, *j*) is used to replace the original proximity function *g*(*k*) involving the constant damping factor *α*. As it was explained earlier, here we can utilize matrix **L**^+^ knowing that it captures all the information we need from the graph. So we define *g* as follows:

$$\begin{array}{c} g\left(k,i,j\right)={\left(\frac{\text{arctan}\left({\left[L^{+}\right]}_{ij}\right)}{\pi }+\frac{1}{2}\right)}^{k} \end{array}$$ (13)

Now let us see how the *S* matrix can be calculated under such a new proximity function. Let us define a matrix *G* where [*G*]_*ij*_ = *g*(1, *i*, *j*). We have:

$$\begin{array}{c} \begin{array}{c} S=I+\left(G\circ P\right)+\left(G\circ G\right)\circ P^{2}+\left(G\circ G\circ G\right)\circ P^{3}+\ldots \end{array} \end{array}$$ (14)

where ∘ is the element-wise Hadamard product operation.

We consider the eigendecomposition of *P* as *P* = *T*Λ*T*^−1^, where Λ is a diagonal matrix whose diagonal entries are eigenvalues and the columns of *T* are the eigenvectors. We have:

$$\begin{array}{c} P^{n}=T\Lambda^{n}T^{-1} \end{array}$$ (15)

and

$$\begin{array}{c} {\left[P^{n}\right]}_{ij}=\sum_{k=1}^{N}T_{ik}{\left(\Lambda_{kk}\right)}^{n}T_{kj}^{-1} \end{array}$$ (16)

Now for each element [*S*]_*ij*_ of matrix *S* we have:

$${\left[S\right]}_{ij}=\overset{\infty }{\underset{l=0}{\sum }}{\left[G\right]}_{ij}^{l}{\left[P^{l}\right]}_{ij}=\overset{N}{\underset{k=1}{\sum }}T_{ik}T_{kj}^{-1}\overset{\infty }{\underset{l=0}{\sum }}{\left({\left[G\right]}_{ij}\left(\Lambda_{kk}\right)\right)}^{l}$$ (17)

$$=\overset{N}{\underset{k=1}{\sum }}T_{ik}T_{kj}^{-1}{\left(1-{\left[G\right]}_{ij}\left(\Lambda_{kk}\right)\right)}^{-1}$$ (18)

Finally to obtain a symmetric matrix for the similarity between each pair of nodes we define $\bar{S}$ as follows:

$$\begin{array}{c} \bar{S}=\frac{1}{2}\left(S+S^{T}\right) \end{array}$$ (19)

**Fig 1:**
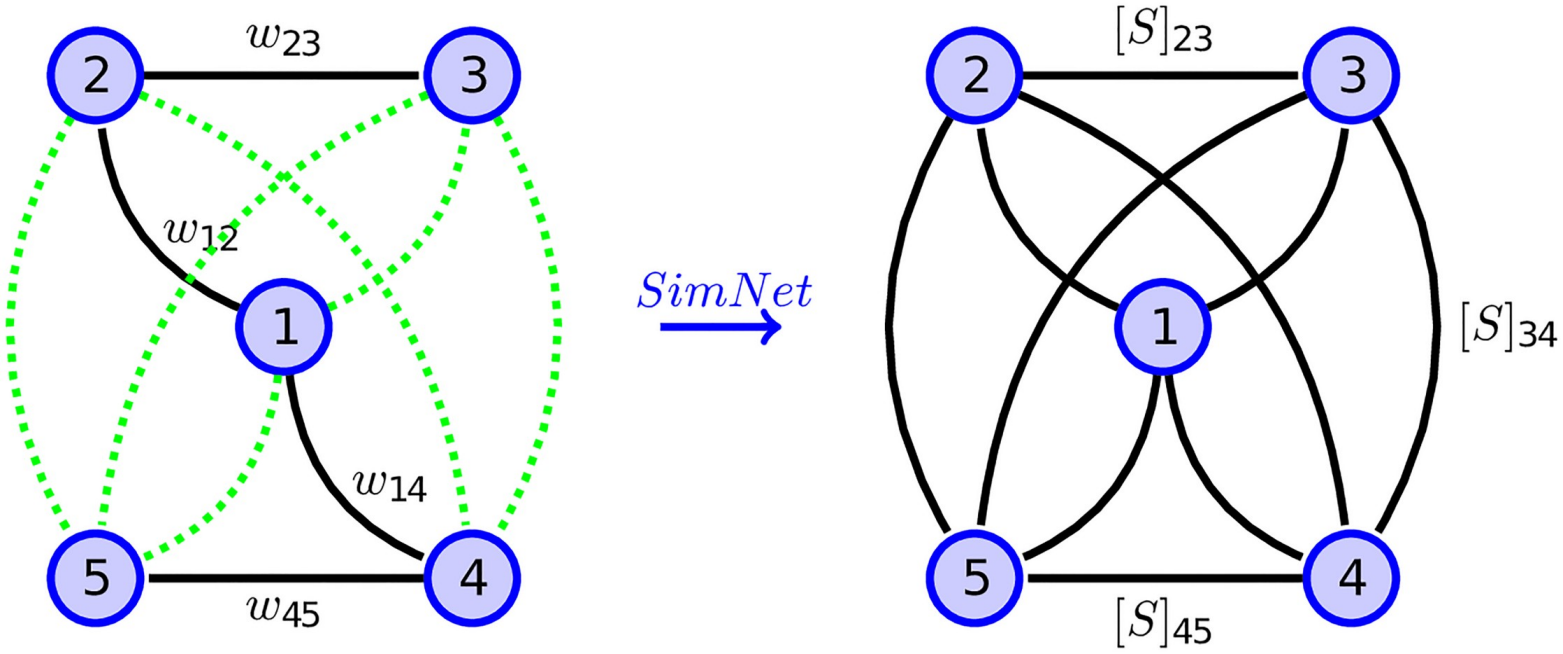
SimNet method. Defining a similarity matrix based on a new way of measuring the closeness between nodes.

This matrix conveys the necessary pair-wise similarity information that is required for building our SimNet model. The matrix also preserves all the properties sufficient for it to reside in a metric space. Namely, identity of indiscernibles, symmetry, and subadditivity are preserved. Hence, one could deduce that it can be utilized to form a transition matrix and in the learning process without the concerns about metric space residency. Our approach essentially performs some refinement to the conventional notion of closeness/proximity for a network. We use Fig 1 to illustrate the high-level ideas. In most conventional approaches, as shown on the left, the closeness measure between two nodes are calculated based on the number of steps required to reach one node from the other. In our approach, however, we introduce a new perspective to measuring the closeness. Our new notion of closeness between two nodes is based on the MCT measure that quantify the average time required for a random walker to reach one node from the other and return back in a principled manner. One can imagine that this is equivalent to say that for any arbitrary two nodes, there is a “virtual edge” that connects them which reveals the strength of closeness between them, as illustrated on the right of the same figure. This allows a richer amount of global structural information to be captured in the above similarity matrix $\bar{S}$.

### 3.2 Learning

Once we have obtained the similarity matrix, we will be able to perform the learning of the embeddings for the nodes based on it. Our first step is to normalize the resulting matrix, arriving at the following new transition matrix *P*_*S*_:

$$D_{S}\;\;=\;\;diag\left[\;{\left[\bar{S}\right]}_{11},{\left[\bar{S}\right]}_{22},\ldots ,{\left[\bar{S}\right]}_{NN}\right]$$ (20)

$$P_{S}\;\;=\;\;D_{S}^{-1}\bar{S}$$ (21)

Based on this matrix *P*_*S*_, we next follow Levy *et al.* [6] and our previous work by Cao *et al.* [9] to define a proper loss function, and learn the node representations via optimization of such a loss function using matrix factorization. We describe the details next.

#### 3.2.1 Loss function

Recall our goal is to learn low-dimensional vector representations for nodes in a network. We slightly abuse the notation here by also using *v*_*i*_ to denote the vector representation of the node *v*_*i*_. Inspired by the negative sampling model by Mikolov *et al.* [5], we define the following loss function for *v*_*i*_:

$$\begin{array}{c} L\left(v_{i}\right)\;\;=\;\;[\;\sum_{v_{j}\in V}{\left[P_{S}\right]}_{ij}\log\sigma \left({\vec{v}}_{i}\cdot {\vec{v}}_{j}\right)\;]\;+\;\lambda E_{v_{j^{\prime }}∼P^{\prime }\left(V\right)}\left[\log\sigma \left(-{\vec{v}}_{i}\cdot {\vec{v}}_{j^{\prime }}\right)\right] \end{array}$$ (22)

where λ is the number of negative samples considered. *P*′(*V*) is a distribution over all nodes in the network. E is the expected value over this distribution. The distribution *P*′(*V*) is defined as:

$$\begin{array}{c} P^{\prime }\left(v_{j}\right)=1/N\sum_{v_{i^{\prime }}\in V}{\left[P_{S}\right]}_{i^{\prime }j} \end{array}$$ (23)

This leads to defining a local loss function over a specific *v*_*i*_ and *v*_*j*_ which we will later apply in the next step for optimization.

$$\begin{array}{ll} l\left(v_{i},v_{j}\right)=\; & \\ & {\left[P_{S}\right]}_{ij}\text{log}\sigma \left({\vec{v}}_{i}\cdot {\vec{v}}_{j}\right)+\lambda /N\underset{v_{i^{\prime }}\in V}{\sum }{\left[P_{S}\right]}_{i^{\prime }j}\text{log}\sigma \left(-{\vec{v}}_{i}\cdot {\vec{v}}_{j}\right) \end{array}$$ (24)

where [*P*_*S*_]_*ij*_ is the relation between *v*_*i*_ and *v*_*j*_. *σ*(.) is the sigmoid function: *σ*(*x*) = (1+*e*^−*x*^)^−1^. The larger the similarity (dot product) between two node representations *v*_*i*_ and *v*_*j*_ is, the higher the value of the sigmoid function. Our goal is to learn such node representations.

#### 3.2.2 Optimization

Our next step is to optimize the loss function. Following [6], to optimize Eq 24, we set ∂*l*/∂*x* = 0 where we define *x* = ${\vec{v}}_{i}.{\vec{v}}_{j}$.

Solving ∂*l*/∂*x* = 0 leads to:

$$\begin{array}{c} e^{x}=\frac{{\left[P_{S}\right]}_{ij}}{\lambda /N\sum_{v_{i^{\prime }}\in V}{\left[P_{S}\right]}_{i^{\prime }j}} \end{array}$$ (25)

Therefore,

$$\begin{array}{c} x=\log(\frac{{\left[P_{S}\right]}_{ij}}{\lambda /N\sum_{v_{i^{\prime }}\in V}{\left[P_{S}\right]}_{i^{\prime }j}}) \end{array}$$ (26)

So since *x* = ${\vec{v}}_{i}\cdot {\vec{v}}_{j}$, we can introduce a matrix *R* with the (*i*, *j*) entry being ${\vec{v}}_{i}\cdot {\vec{v}}_{j}$ as defined in the above equation:

$$\begin{array}{c} {\left[R\right]}_{ij}=\log(\frac{{\left[P_{S}\right]}_{ij}}{\lambda /N\sum_{v_{i^{\prime }}\in V}{\left[P_{S}\right]}_{i^{\prime }j}}) \end{array}$$ (27)

Following the work of Levy *et al.* [6], to reduce noise, we consider the non-negative matrix *R*^+^ as follows:

$$\begin{array}{c} R_{i,j}^{+}=\max\left(0,R_{i,j}\right) \end{array}$$ (28)

Amongs various methods for matrix factorization we choose to perform singular value decomposition (SVD) [31] due to its simplicity and effectiveness as shown in our previous work [9]. For a given matrix *R*^+^, its SVD yields the following:

$$\begin{array}{c} R^{+}=U\Sigma V^{T} \end{array}$$ (29)

where *U* and *V* are orthogonal matrices from the space $R^{N\times N}$, and Σ = *diag*(*σ*_1_, …, *σ*_*N*_) is a diagonal matrix whose diagonal entries are singular values of *R*^+^, satisfying: *σ*_1_ ≥ *σ*_2_ ≥ …*σ*_*n*_ ≥ 0.

Our primary purpose in this paper is to represent low-dimensional vectors for a network’s nodes. Therefore we use an alternative matrix $R_{d}^{+}\in R^{d\times d}$ which is a low-rank approximation of *R*^+^, defined as follows:

$$\begin{array}{c} R_{d}^{+}=U_{d}\Sigma_{d}V_{d}^{T} \end{array}$$ (30)

where *U*_*d*_ and *V*_*d*_ are matrices constructed from the first *d* columns of matrices *U* and *V*, and Σ_*d*_ is a diagonal matrix constructed from its first *d* singular values: *diag*[*σ*_1_, …, *σ*_*d*_].

The above leads to factorization of matrix *R*^+^ to two separate matrices which we call *W*_1_ and *W*_2_.

$$\begin{array}{c} R^{+}\approx W_{1}W_{2} \end{array}$$ (31)

where *W*_1_ = *U*_*d*_(Σ_*d*_)^1/2^ and $W_{2}={\left(\Sigma_{d}\right)}^{1/2}V_{d}^{T}$.

The *i*-th blackrow of *W*_1_ gives us the final vector representation of the *i*-th node *v*_*i*_ in the network.

## 4 Experiments

In this section we conduct experiments on several real-world datasets to assess the effectiveness of the chosen similarity measure and our graph representation method. To understand how effective our method is in general, we conduct experiments on four different tasks where three different types of networks are involved. These datasets include a language network, a social network and a citation network. We also compare our method against various existing baseline approaches.

### 4.1 Experiment Setup

#### 4.1.1 Datasets

We provide a brief discussion on the three types of datasets that we use for our experiments. In Table 1, we list down the detailed summaries of such datasets.

**Table 1:** Statistics of the real-world networks.

	Social Network	Language Network	Citation Network
Name	**Blogcatalog**	**20-NewsGroup (200 samples)**	**DBLP (author citation)**
Type	unweighted	weighted	wighted
#(*V*)	10,312	600, 1200 and 1800	7,314
#(*E*)	333,983	Fully connected	72,927
Avg. degree	64.78	—	19.94
# Labels	39	3, 6 and 9	3
Task	Classification & Link Prediction	Clustering	Visualization

***Blogcatalog*** is a social network. Each node indicates a blog author and each edge demonstrates the relation between two bloggers. The labels show the different topics which the bloggers talk about. We will use this dataset to conduct experiments in a supervised setting, where we consider the multi-label classification task using the learned node representations. Moreover, Blogcatalog network will be used in a link prediction task where we score the possibility of a link existing between each pair of nodes given their embeddings.

***DBLP Network*** is a citation network. Each node indicates an author and each edge between two authors has a weight illustrating the number of citations from one to another. First we choose to look at authors from six popular conferences. Next we group them into three categories: *1. data mining*, *2. computer vision*, and *3. machine learning*. Specifically, *WWW* and *KDD* are from the first category, *CVPR* and *ICCV* belong to the second category and *NIPS* and *ICML* fall into the third category. This is a weighted network that will be used for the visualization task.

***20-Newsgroups*** is a language network with approximately 20,000 different newsgroup documents from 20 categories. Each word in each document is represented by its tf-idf score which together build up a vector for the whole document. Each document is then regarded as a node in such a language network, and the weight over an edge between any two node is defined as the cosine similarity between their respective vectors. We follow [32] to randomly sample 200 documents from a topic and form three networks from 3, 6 and 9 different newsgroups. Specifically,

**3-Newsgroups** includes the following news categories:

*comp.graphics, comp.graphics*, and *talk.politics.guns*

**6-Newsgroups** includes:

*comp.sys.mac.hardware, rec.motorcycles, rec.sport.hockey*,

*soc.religion.christian, alt.atheism*, and *omp.sys.ibm.pc.hardware*

**9-Newsgroups** includes:

*talk.politics.mideast, talk.politics.misc, comp.os.mswindows.misc*,

*sci.crypt, sci.med, sci.space, sci.electronics, misc.forsale*, and

talk.religion.misc

These are weighted and also fully-connected networks which will be used for performing clustering tasks.

#### 4.1.2 Baseline methods

In this section, we will first compare our choice of MCT measure with other popular similarity measures which quantify the global similarities between nodes in a network. Next, after recognizing the most efficient candidate among similarity measures and justifying our original choice, we will move on to evaluate the performance of MCT-based SimNet against previous network embedding approaches.

**Similarity measure variants**. We choose three other well-known similarity measures to replace the MCT measure used in Section 3.1. The learning process is then carried out on these variants of SimNet.

1. **Personalized PageRank**: Personalized PageRank (PPR) [33] is a common similarity measure among nodes, practically used for graph mining tasks. We call this variant **SimNet-PPR**.
2. **Maximal Entropy Random Walk**: Maximal Entropy Random Walk (MERW) [34] is based on the nodes tendency to be linked to central nodes in a network and tries to model this behaviour. This method aims to maximize the entropy rate of the random walk. This approach will be referred to as **SimNet-ME**.
3. **Katz index**: Katz [26] is an index which sums the influence of all present paths between each pairs of nodes while penalizing paths by their length. When using this index as our similarity measure, we call the resulting approach **SimNet-Katz**.

In the rest of the paper, **SimNet** refers to the default version of our method which utilizes MCT as the similarity measure. Next, we consider the following 5 previous approaches for network embedding as our baselines.

1. **Spectral Clustering**: spectral clustering [35] is an algorithm that aims at minimizing normalized cut (NCut). It also uses matrix factorization methods, but it focuses on a different matrix—the Laplacian matrix.
2. **DeepWalk**: DeepWalk [7] is an approach for learning latent representations of nodes in a network using local information obtained from truncated random walks. It is originally only applicable to unweighted networks.
3. **LINE**: LINE [8] is a method for learning graph representations on large-scale information networks. Its loss function is based on first-order and second-order relational information between each pair of nodes among the network. The model can also make use of certain higher-order proximity information by using an extended neighborhood. The learning procedure involves several steps. In the first step, it learns the first *d* dimensions by performing a BFS-style sampling over first-order neighbors. This is followed by the second step, where it samples nodes strictly at a 2-hop distance for learning the next *d* dimensions.
4. **GraRep**: GraRep [9] is our recently proposed method for learning node representations of weighted graphs. It captures the *k*-th order proximity information between a node and its *k*-th order neighbors using the skip-gram model. GraRep constructs its representation of the graph by concatenating the results obtained from each step. However the value *k* can not be any arbitrarily large number and is usually empirically set to less than 6. Increasing this value linearly will also lead to a linear increasing of the resulting node representations’ dimensions. The dimension of the resulting learned node representations will grow linearly as we increase the value *k*.
5. **node2vec**: node2vec [10] is a recent approach for learning representations for nodes in networks. It maps nodes to a low-dimensional space of features. node2vec designs an objective that seeks to preserve local neighborhoods of nodes. The objective is then optimized using stochastic gradient descent (SGD). A second order random walk approach is applied to generate neighborhoods for nodes.

#### 4.1.3 Parameter settings

Following [8, 9], we set the dimension (dim) of representations to 128 and report results for the Blogcatalog network, so as to make a fair comparison. For the two previous models LINE and GraRep, final representations are constructed by concatenating smaller vector representations. We thus also report the results when the dimension of the smaller vectors are set to 128 (for GraRep, we set *k* = 6, following [9]).

For the clustering experiments on the 20-Newsgroup dataset, we report results under 3 different dimensions for the representations: 64 (which was used in [32]), 192 (which is 64 × 3—the dimension that leads to the optimal results for GraRep), and 72 (which yields the best performance for SimNet).

There are also some model-specific parameters. For DeepWalk, we followed [7] to set the window size to 10 and the walk length to 40. For LINE, as suggested in [8], we set the order to 2 so that both first-order and second-order proximity information can be concatenated to form the representations. We also employed the reconstruction strategy for nodes with small degrees so as to achieve the optimal performance. First, it learns *d* dimensions by BFS-style simulations over immediate neighbors of nodes which is then followed by learning the next *d* dimensions by sampling nodes strictly at a 2-hop distance from the source node. It eventually yields representations with a total dimension of 2*d*. For GraRep, as suggested in [9], we set the parameter *β* to 1/*N*. We used the following combinations of *k* and *d* to obtain representations of different dimensions (note that *dim* = *kd*): we set *k* = 4 and *d* = 16 for *dim* = 64, *k* = 4 and *d* = 32 for *dim* = 128, then *k* = 3 and *d* = 24 for *dim* = 72, and as suggested in [9], we set *k* = 3 and *d* = 64 for *dim* = 192. For node2vec, we followed their paper [10] to set the window size and the number of walks per node to 10, while the length of random walk is set to 80. We tune different values for *p* and *q* to get the best performance, which is when *p* = *q* = 0.25. We also repeat the experiments for 10 random seed initializations.

For different variants of SimNet, we choose the corresponding damping factors to reach the best performance. This leads us to the choice of *α* = 0.85 for SimNet-PPR and SimNet-ME while SimNet-Katz performs best with *α* = 0.5.

**SimNet-c**. For our SimNet model, to understand the effectiveness of using MCT as the closeness measure, we also developed a different, simplified version of our model. Specifically, this version uses a similarity matrix with a constant dumping factor *α*, which we will call **SimNet-c**. Hence, there will be no dynamic damping factor involved which allows us to better compare and capture the power of MCT-based damping factor. We make a rational choice by selecting *α* = 0.5 in our experiments for the SimNet-c model. Our experiments show that although increasing *α* can lead to slightly better results for the clustering tasks, doing so at the same time leads to much worse results in the classification task. So to have a well-made decision we choose *α* = 0.5. Our main model **SimNet** utilizes MCT-based damping factors as described in this paper to calculate the similarities between nodes.

### 4.2 Tasks and results

In this section we empirically demonstrate the effectiveness of our SimNet model by using the learned representations for performing classification, clustering, visualization, and link prediction tasks on real-world networks. We make the source code of SimNet available at https://github.com/Moein-Khajehnejad/SimNet/.

#### 4.2.1 Classification

The first experiment is conducted on a multi-label classification task. Our focus is on a supervised classification task for a social network such as Blogcatalog, where we classify different nodes into different classes by using their learned node representations as input features. We use the LibLinear package [36] which is an efficient implementation of linear classifiers that is capable of handling data with millions of instances and features. During training, a certain fraction of nodes (with labels) are selected to form the training set. Next the task is to predict the labels of the remaining nodes. We use one-vs-rest logistic regression as the classification method, and repeat the experiments 10 times and report the averaged Micro-F1 and Macro-F1 scores. We perform this task for randomly selected samples of 10 to 90 percent of the nodes in the network which are used for training and in each round the rest of the nodes are used for prediction and evaluation. The results are reported in Table 2. While SimNet proves to be consistently better than all its variants, we also evaluate its performance against previously introduced network embedding baselines. We can see that when the Macro-F1 score is considered, node2vec and SimNet perform the best both giving really satisfying scores compared to the rest. As the training set increases, node2vec starts to yield better results under this Macro-F1 though SimNet is still very close and performs almost as efficiently. The better results of node2vec under this metric indicate its better predictive power for rare classes. However, when Micro-F1 is considered, the results show that our proposed SimNet consistently yields the best scores across different setups. From the experiments we can also observe that the simpler version SimNet-c, which makes use of a constant closeness measure, consistently yields lower results. Our results (even SimNet-c) are also better than GraRep, which we believe can be largely due to its ability to capture rich higher-order proximity information.

**Table 2:** Results on Blogcatalog.

Metric	Algorithm	10%	20%	30%	40%	50%	60%	70%	80%	90%
Micro-F1	SimNet (*dim* = 128)	**39.61**	**42.22**	**42.98**	**43.95**	**44.47**	**45.01**	**45.32**	**45.40**	**46.31**
	SimNet-c (*dim* = 128)	38.54	41.12	42.40	43.28	43.67	44.12	44.23	44.46	45.03
	SimNet-PPR (*dim* = 128)	37.95	40.36	41.50	43.56	43.94	44.51	44.82	45.03	45.51
	SimNet-ME (*dim* = 128)	35.87	38.36	39.44	40.45	40.93	41.19	41.32	41.68	42.72
	SimNet-Katz (*dim* = 128)	38.11	40.93	42.09	42.91	43.35	43.87	43.96	44.18	44.61
	node2vec (*dim* = 128)	35.56	38.72	40.32	41.33	42.11	42.73	42.85	43.00	43.59
	GraRep (*k* = 4, *d* = 32, *dim* = 128)	35.61	39.98	41.47	42.12	42.38	43.58	42.74	43.08	43.20
	GraRep (*k* = 6, *d* = 128, *dim* = 768)	38.31	40.44	41.29	41.92	42.51	42.96	43.54	43.61	44.33
	LINE (*d* = 64, *dim* = 128)	33.98	37.49	38.05	39.76	40.51	41.21	41.48	41.89	42.02
	LINE (*d* = 128, *dim* = 256)	37.12	39.57	40.71	41.66	42.27	42.63	43.11	43.44	43.79
	DeepWalk (*dim* = 128)	36.22	38.71	39.75	40.79	41.28	41.54	41.67	41.70	42.83
	Spectral Clustering (*dim* = 128)	37.23	39.33	40.39	40.76	41.32	41.47	41.52	41.65	42.16
Macro-F1	SimNet (*dim* = 128)	**23.83**	**27.88**	**29.63**	30.71	31.50	**32.43**	32.66	32.92	33.67
	SimNet-c (*dim* = 128)	23.07	27.21	28.61	29.48	30.27	30.80	30.92	31.53	31.77
	SimNet-PPR (*dim* = 128)	22.84	26.73	28.44	30.12	30.68	31.12	31.55	31.93	32.26
	SimNet-ME (*dim* = 128)	19.87	22.98	24.02	24.57	25.29	26.07	26.88	27.25	27.89
	SimNet-Katz (*dim* = 128)	22.55	26.91	28.24	29.28	30.19	30.68	30.75	31.44	31.59
	node2vec (*dim* = 128)	23.44	26.38	29.16	**30.85**	**31.76**	32.18	**32.73**	**33.19**	**33.80**
	GraRep (*k* = 4, *d* = 32, *dim* = 128)	20.81	24.89	27.33	27.95	28.43	28.78	28.94	29.03	29.31
	GraRep (*k* = 6, *d* = 128, *dim* = 768)	23.13	25.68	26.58	27.72	28.33	28.90	29.59	30.08	30.89
	LINE (*d* = 64, *dim* = 128)	16.23	18.99	21.12	21.57	21.85	22.01	22.35	22.68	22.96
	LINE (*d* = 128, *dim* = 256)	19.71	22.92	24.67	25.54	26.38	27.34	28.03	28.89	29.52
	DeepWalk (*dim* = 128)	21.47	23.42	24.39	24.93	25.69	26.56	27.08	27.22	28.14
	Spectral Clustering (*dim* = 128)	19.15	22.38	23.44	24.46	24.91	25.07	25.41	25.63	26.49

#### 4.2.2 Clustering

To assess the effectiveness of SimNet in a clustering task and compare it to the other baselines, following the previous research effort [9], we evaluate and report the average *normalized mutual information* (NMI) score [37] over 20 consecutive runs for all baselines and all three groups of 20-Newsgroups network. This is a network with each node having a single label and having an overall number of three classes. The results are shown in Table 3. When the commonly used setting *dim* = 64 is selected, our SimNet model returns the best results. When *dim* = 192 is used, GraRep performs better than our model on the 3NG dataset, but our model is better on 6NG and 9NG datasets. Our model achieves the optimal results when the setting *dim* = 72 is used, under which it also outperforms all previous approaches. SimNet clearly holds its stronger position against all its variants in this task as well. The relative effectiveness of each model was also reported in the previous work [9].

**Table 3:** Clustering results on 20-newsgroups (200 samples).

Algorithm	*dim* = 64			*dim* = 72			*dim* = 192		
	3NG	6NG	9NG	3NG	6NG	9NG	3NG	6NG	9NG
SimNet	**78.62**	**67.77**	**59.55**	**79.34**	**68.63**	**60.19**	78.86	**68.57**	**59.97**
SimNet-c	76.87	65.93	57.18	77.27	67.39	58.55	76.95	68.08	59.61
SimNet-PPR	69.24	65.37	49.21	73.14	66.81	59.32	70.81	68.35	55.74
SimNet-ME	50.33	51.46	47.28	46.91	50.73	46.18	58.25	50.84	46.92
SimNet-Katz	76.62	65.68	57.76	77.01	67.13	58.29	76.73	68.64	59.30
GraRep	76.25	65.51	56.03	77.82	66.28	57.74	**81.12**	67.54	59.39
node2vec	22.17	18.86	16.33	21.31	18.79	15.12	16.75	15.55	14.03
LINE	75.24	63.99	53.62	76.41	64.78	54.10	79.25	63.05	52.17
DeepWalk	65.64	63.67	48.89	65.16	62.98	47.56	60.88	60.04	47.14
Spectral Clustering	49.12	51.03	46.93	46.37	50.43	39.13	28.46	27.81	35.98

We also note that the representations produced by node2vec do not perform well for this clustering task. We believe this is partly because the biased random walk employed by node2vec as discussed in their paper does not fit the network topology that we consider in this task very well. To get a better insight, consider a walker that just arrived at node *v* from *t* and now has to decide on the next step. Since 20-Newsgroup is a fully-connected network the distance between *t* and every other possible node is always 1, unless the walker is returning to the source node *t*. This essentially makes their in-out parameter *q* vacuous (which is used to bias the walker towards exploring nodes which are closer to or further away from *t*). Applying node2vec on such a graph leads to a matrix no different than the original transition matrix itself with just its diagonal entries being changed. Also because of the very large number of choices the walker has in each step (since all the nodes are at distance 1 from the source and there are no neighbours of second order or more), node2vec requires more time to surf the network while performing the BFS strategy to form the set of neighbours.

The simpler version of our model SimNet-c, that makes use of higher-order proximity information of the graph, performs competitively when compared with the previous model GraRep. By using the MCT-based proximity function, our full model SimNet significantly outperforms SimNet-c.

#### 4.2.3 Visualization

After SimNet proved to be most efficient in its default form based on an MCT similarity measure, we will exclude its variants from the results in the remaining tasks as they fall short in providing competing results.

In this experiment we aim to visualize the learned representations by examining a real citation network based on DBLP. We use the standard t-SNE toolkit [38] and get a visualization of the learned graph representations. A 2-dimensional visualization of the graph is obtained with each colored dot as an indicator of each node. As mentioned before, we categorize the data of the DBLP network into three main groups, and the nodes belonging to the same group share the same color. This task also gives us the Kullback-Leibler divergence indicating the error between the pairwise similarities of the input and their corresponding projection in the resulting 2-dimensional mapping. Therefore a lower KL divergence indicates a better network representation.

**Fig 2:**
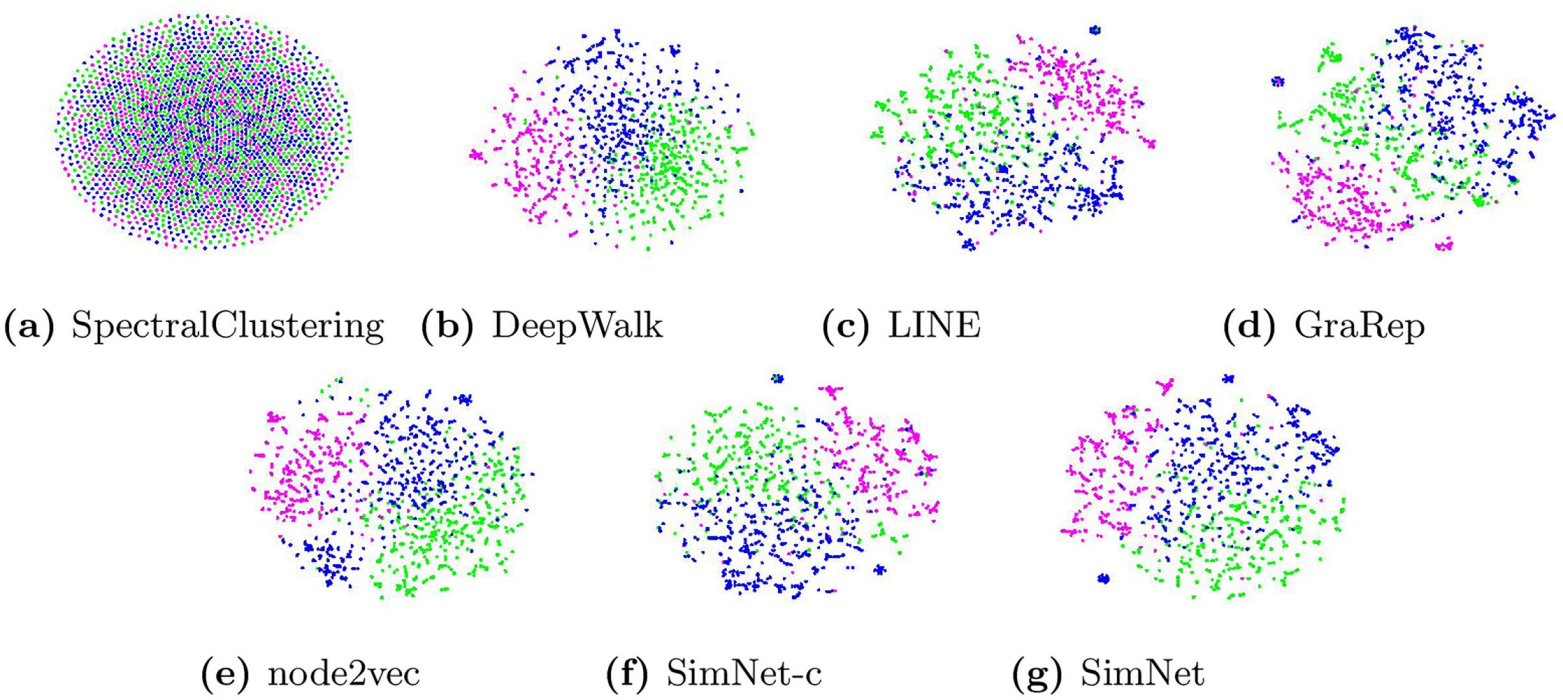
Visualization of author citation network. Each point indicates one author. Green: *Data Mining*, magenta: *Computer Vision* and blue:*Machine Learning*.

As it can be observed from Fig 2, in Spectral Clustering the visualization is not very informative since the nodes of different groups with different colors are heavily mixed together. For both DeepWalk and node2vec there are better groupings of similar nodes, but the boundaries between the groups are still not very clear and nodes of the same color can appear at different regions to form different groups. LINE, GraRep, SimNet-c and SimNet give better results with much more precise borders between different groups, with SimNet performing the best and having the smallest KL-divergence, as shown in Table 4.

**Table 4:** Final KL divergence for the DBLP dataset.

Algorithm	SimNet	SimNet-c	node2vec	GraRep	LINE	DeepWalk
KL divergence	**0.9193**	0.9385	1.1233	0.9371	1.0054	1.0804

#### 4.2.4 Link Prediction

**Fig 3:**
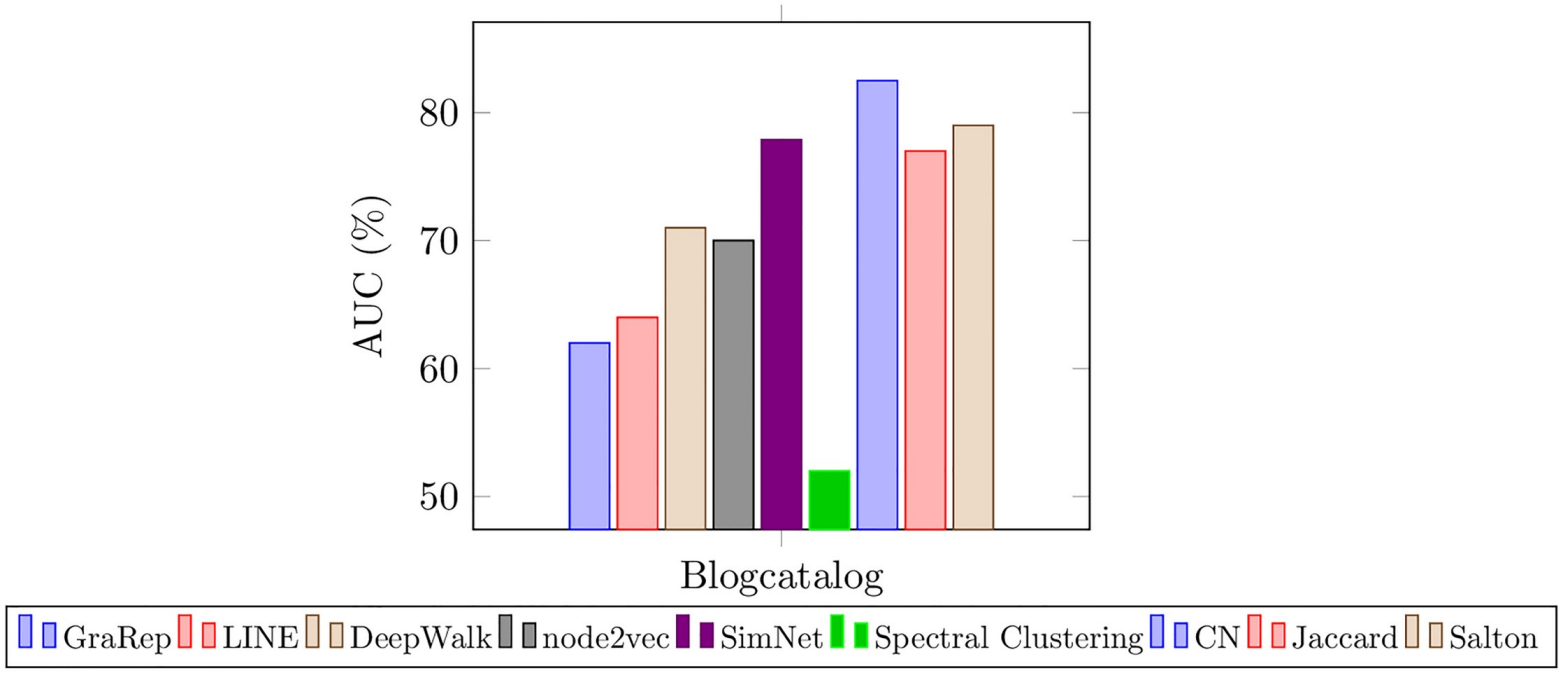
Experimental results on link prediction.

In this last experiment, the goal is to predict the existence of an edge between any given pair of nodes. This task will reveal the edge predictability power of SimNet and the baseline methods. For this cause, we randomly hide 20% of connected node pairs and simultaneously the same number of node pairs from the disconnected ones in the Blogcatalog graph. These nodes will be our test set. The rest of the data are shuffled and the remaining connected node pairs together with an equal number of remaining disconnected pairs are kept for training. Next, the obtained representation vectors from different embedding modalities will be employed in a logistic regression method to predict the probability of edge existence for a given node pair [39]. Finally, the trained link prediction model is applied on the test set and the results are shown in Fig 3. We measure the AUC value which indicates the probability that the score of an unobserved edge is higher than that of a nonexistent one for SimNet as well as the baseline methods. In this specific task, we also take into account three most commonly exploited link prediction baseline: Common Neighbors (CN), Jaccard Index and Salton Index [40]. As illustrated in Fig 3, SimNet’s link prediction power exceeds GraRep, node2vec, LINE, DeepWalk, and Spectral Clustering however, CN outperforms all the rest with the highest AUC score in this task.

### 4.3 Parameter sensitivity

**Fig 4:**
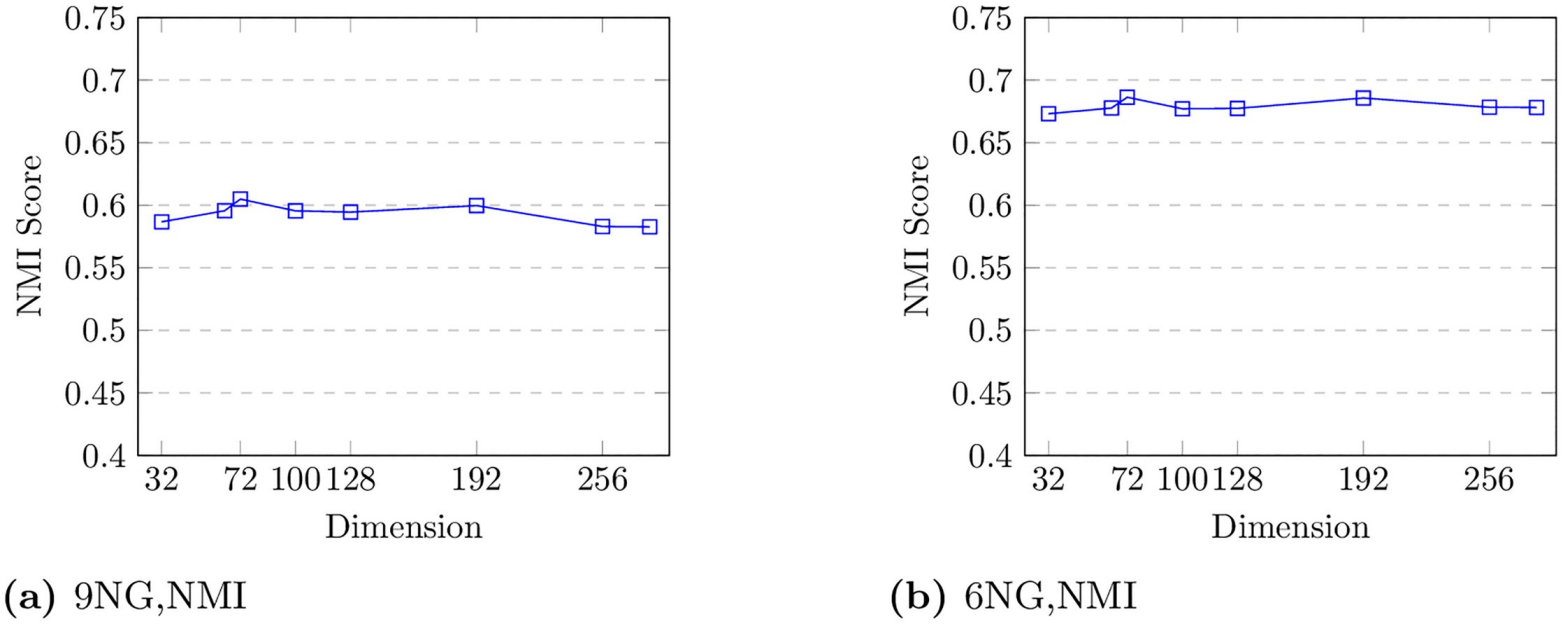
Performance of SimNet over different dimensions.

In this section we will discuss the effect of varying our model’s parameters. Specifically we will investigate how different choices of dimension can affect the results and also the running time of our proposed method. Fig 4 shows the clustering performance of SimNet under different dimension settings. We can see that when dimension is small, increasing the dimension can lead to an increase in the NMI score for 20-Newgroups network, and in our experiments we get the best results when dimension is chosen as 72. However a dimension larger than 72 will not make any significant improvements in the NMI score. These results demonstrate the fact that with a larger dimension we can gather complementary information of the network. However for a *d* greater than 72 no significant additional information can be obtained.

**Fig 5:**
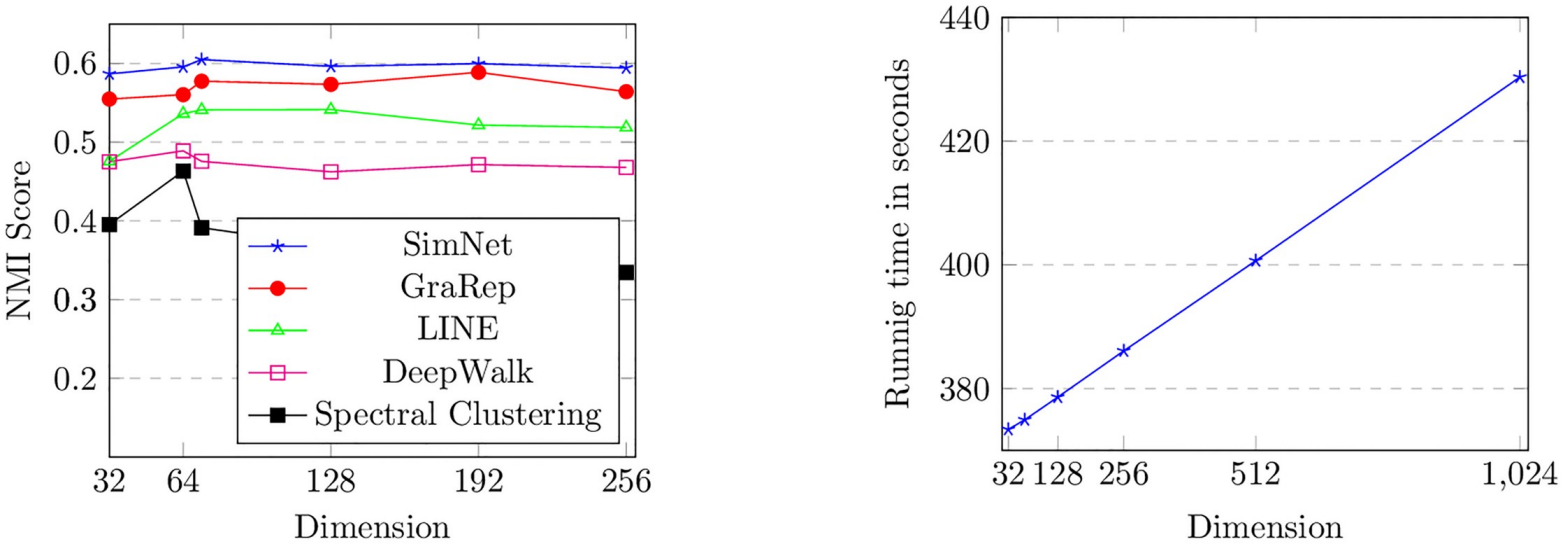
Left: Performance of different methods over different dimensions for 9NG network. Right: Running time.

**Fig 6:**
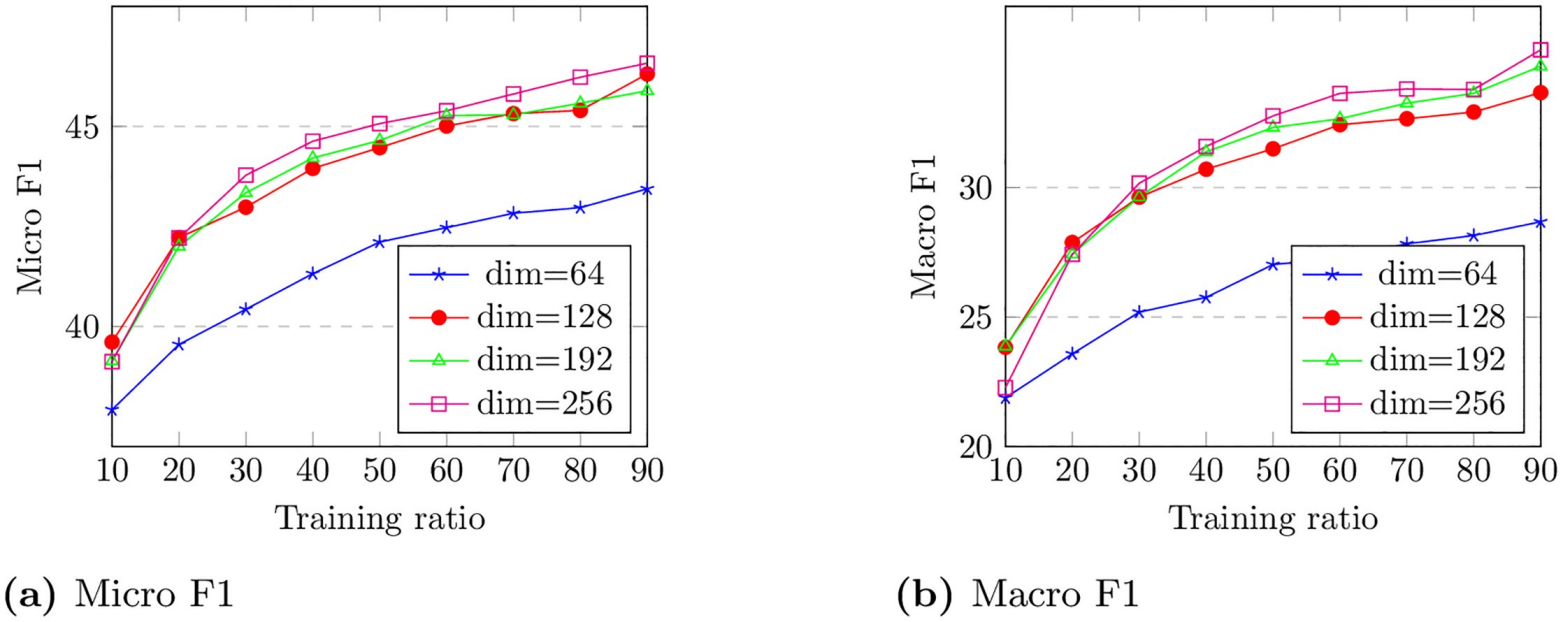
Performance on Blogcatalog over training ratio.

In the left part of Fig 5, we compare the NMI scores of the SimNet model and other baselines when the dimension changes. It illustrates that either in a large or small dimension SimNet consistently outperforms other baselines with representations under the same dimension. As it can be seen in the right part of Fig 5, running time of the method increases approximately linearly as the dimension increases. In Fig 6 we have measured and plotted Micro-F1 and Macro-F1 scores for varying sizes of training sets and different dimensions in Blogcatalog network. We observed that an increase in the dimension will give us an even better performance alongside the fact that this increase will not cost us much time since the right part of Fig 5 shows us an approximately linear trend.

Finally a complexity comparison is performed among the main competing methods in Table 5. In this tabel, *d* is the embedding dimensionality, *n* the number of nodes, *m* the number of edges, and s the number of samples used, and *t* the number of iterations in GraRep.

**Table 5:** Comparison of network embedding methods in terms of complexity in time.

Method	SimNet	node2vec	GraRep	LINE	DeepWalk	Spectral Clustering
Time Complexity	$O(n^{3})$	O(dsn)	$O(tn^{3})$	O(dsn)	O(dnlogn)	$O(n^{3})$

## 5 Conclusion

In this paper we proposed SimNet, a novel model for learning graph representations using latent structural information of the network. For the first time we utilized the mean commute time (MCT) measure in the learning process of network embeddings. Instead of using a conventional method involving simple constant factors for measuring closeness between nodes, we introduce the use of MCT as such a measure. By calculating MCT, we show that we are able to adequately quantify the closeness measure between nodes in a network in a principled manner, which can then be used to derive a better similarity matrix for learning network representations. By replacing MCT with other popular similarity measures when building the similarity matrix which is later used in the learning process, we prove that the well-chosen MCT-based similarity measure clearly yields better results.

We empirically demonstrate the effectiveness of our approach through extensive experiments across different tasks. One of the current challenges that we are facing is the computation of **L**^+^—the pseudoinverse of the Laplacian matrix involved in the learning process is expensive. In our future work we plan to optimize the computation of **L**^+^, and explore simple yet precise approximations of this measure for learning improved network embeddings.
