# Correction: SimNet: Similarity-based network embeddings with mean commute time

**DOI:** 10.1371/journal.pone.0230096

**Published:** 2020-03-02

**Authors:** Moein Khajehnejad

An additional affiliation is missing for the first author. Moein Khajehnejad is also affiliated with: Faculty of Information Technology, Monash University, Melbourne, Australia.

The corresponding author’s email address is incorrect. Dr. Khajehnejad 's correct email address is: Moein.khajehnejad@monash.edu.
